# Selective suppression of the α isoform of p38 MAPK rescues late-stage tau pathology

**DOI:** 10.1186/s13195-016-0221-y

**Published:** 2016-12-15

**Authors:** Nicole Maphis, Shanya Jiang, Guixiang Xu, Olga N. Kokiko-Cochran, Saktimayee M. Roy, Linda J. Van Eldik, D. Martin Watterson, Bruce T. Lamb, Kiran Bhaskar

**Affiliations:** 1 Department of Molecular Genetics and Microbiology, MSC08 4660, 1 University of New Mexico, University of New Mexico, Albuquerque, NM 87131 USA; 2 Stark Neurosciences Research Institute, Indiana University, 320W 15th Street, NB Suite 414C, Indianapolis, IN 46202 USA; 3 Department of Neurosciences, The Ohio State University, 4198 Graves Hall, 333 West 10th Avenue, Columbus, OH 43210 USA; 4 Department of Pharmacology, Northwestern University Feinberg School of Medicine, Ward Building Room Mail Code W896, 303 E Chicago Avenue, Chicago, IL 60611 USA; 5 Sanders-Brown Center on Aging, University of Kentucky, 101 Sanders-Brown Bldg., 800S. Limestone Street, Lexington, KY 40536 USA

**Keywords:** p38 mitogen activated protein kinase, Alzheimer’s disease, tau, tauopathies, hTau, SB239063, p38 MAPK inhibitor, MW01-10-181SRM, MW181 and MK2 deficiency

## Abstract

**Background:**

Hyperphosphorylation and aggregation of tau protein are the pathological hallmarks of Alzheimer’s disease and related tauopathies. We previously demonstrated that the microglial activation induces tau hyperphosphorylation and cognitive impairment via activation of p38 mitogen-activated protein kinase (p38 MAPK) in the hTau mouse model of tauopathy that was deficient for microglial fractalkine receptor CX3CR1.

**Method:**

We report an isoform-selective, brain-permeable, and orally bioavailable small molecule inhibitor of p38α MAPK (MW181) and its effects on tau phosphorylation in vitro and in hTau mice.

**Results:**

First, pretreatment of mouse primary cortical neurons with MW181 completely blocked inflammation-induced p38α MAPK activation and AT8 (pS199/pS202) site tau phosphorylation, with the maximum effect peaking at 60–90 min after stimulation. Second, treatment of old (~20 months of age) hTau mice with MW181 (1 mg/kg body weight; 14 days via oral gavage) significantly reduced p38α MAPK activation compared with vehicle-administered hTau mice. This also resulted in a significant reduction in AT180 (pT231) site tau phosphorylation and Sarkosyl-insoluble tau aggregates. Third, MW181 treatment significantly increased synaptophysin protein expression and resulted in improved working memory. Fourth, MW181 administration reduced phosphorylated MAPK-activated protein kinase 2 (pMK2) and phosphorylated activating transcription factor 2 (pATF2), which are known substrates of p38α MAPK. Finally, MW181 reduced the expression of interferon-γ and interleukin-1β.

**Conclusions:**

Taken together, these studies support p38α MAPK as a valid therapeutic target for the treatment of tauopathies.

## Background

Alzheimer’s disease (AD), a progressive neurodegenerative condition, is diagnosed in 60–80% of total dementia cases and presents with memory loss and other cognitive impairments. AD is the sixth leading cause of death in the United States. Neurofibrillary tangle (NFT) pathology, one of the major pathological hallmarks of AD and related tauopathies, occurs when microtubule-associated protein tau (MAPT or tau) undergoes hyperphosphorylation and aggregates as NFTs. Recent studies suggest that NFT pathology closely correlates with neurodegeneration and cognitive decline [1, 2].

Over the years, studies have suggested that one of the major functions of tau is to bind and stabilize microtubules. However, immune-mediated or genetic-mediated depletion of tau did not seem to affect microtubule stability in vitro and in vivo, suggesting the possibility of non-microtubule binding functions of tau [3, 4]. Indeed, our previous studies have suggested that the PXXP motif of tau can interact with SH3 domains of the Src family kinases (such as Fyn and Src) [5–7], which in turn affect actin remodeling in growth cones [7]. These tau–fyn [8] and tau–actin [9] (also reviewed in [10]) interactions were also demonstrated in earlier studies. The non-microtubule binding functions of tau are equally important because a recent study demonstrated that tau has a very short interaction time with microtubules in physiological conditions [11]. Based on these studies, the current consensus in the field is that tau can contribute to the disease process either via loss of function (e.g., failure to bind to microtubules) or gain of toxic function (e.g., by affecting various types of non-microtubule binding functions). In further support of this, our previous studies have demonstrated that phosphorylation or disease-related point mutations in tau affect tau–Fyn interactions and may contribute to pathogenesis [12, 13]. Therefore, phosphorylation of tau by various Ser/Thr or Tyr kinases may directly impact on both microtubule-binding and gain of toxic functions of tau, which may contribute to neurodegeneration in AD [14, 15] (reviewed in [16, 17]). Most of these tau phosphorylation sites are considered to be targeted by more than one kinase [18]. In fact, there are currently more than 20 serine/threonine kinases reported to phosphorylate tau in vitro: glycogen synthase kinase 3 beta (GSK-3β), cyclin-dependent kinase 5 (Cdk5), microtubule affinity regulating kinase 1–4 (MARK1–MAPK4), extracellular signal-related kinase (ERK), p38 mitogen-associated protein kinase alpha (p38α MAPK), and c-Jun N-terminal kinase (JNK) are the best examples [19–21]. However, their direct involvement in AD remains elusive and those kinases amenable to therapeutic intervention are not yet evident.

We have demonstrated recently that activated microglia induced tau hyperphosphorylation via activation of the p38α MAPK signaling pathway in the hTau mouse model of tauopathy [22, 23]. Notably, we observed elevated levels of active (phosphorylated) p38α MAPK, but not GSK3β or regulators (p35/p25) of Cdk5, correlating with increased tau phosphorylation in the hippocampus of hTau mice deficient for the chemokine receptor CX3CR1 [22]. This is consistent with a previous in vitro study in which neocortical neurons pretreated with the popular-mixed kinase inhibitor SB203580 and then treated with interleukin-1β (IL-1β) showed significantly reduced tau phosphorylation and restored synaptophysin levels [24]. Signaling through p38 MAPK, especially the p38α isoform, has also been discovered recently to be a critical component for cytokine production in activated microglia [25, 26]. The p38α-mediated cytokine overproduction in microglia has been linked to inflammation-induced neurotoxicity in a variety of mouse models [22, 27–33]. These studies suggest that targeting the p38α MAPK signaling pathway should be tested as a potential strategy to suppress microglial innate immune responses and prevent inflammation-mediated tau pathology.

The p38α MAPK has been a drug target in many animal models of disease, including peripheral inflammatory disorders (e.g., asthma and rheumatoid arthritis) and central nervous system (CNS) disorders. A variety of small-molecule p38α MAPK inhibitors have been tested in preclinical animal models of CNS diseases. These inhibitors vary in their target specificity, which raises concerns about linkage of target modulation to phenotype—a key aspect of drug discovery target validation, as documented previously [34]. For example, first-generation p38α MAPK inhibitors such as MW01-2-069A-SRM were used in a mouse model of AD-relevant pathophysiology that involves intracerebroventricular (ICV) infusion of oligomeric Aβ_1–42_ [35]. Other studies used commercially available mixed kinase inhibitors such as SB203580, SB239063, and FR167653 in models of amyotrophic lateral sclerosis [32], contusion model of spinal cord injury [33], controlled cortical impact model of traumatic brain injury [36], cerebral ischemia [37], neuropathic pain in a rat model of lumbar disc herniation [38], cerebral ischemia in a rat model of stroke [39], and Parkinson’s disease in MPTP-treated mice [40]. While kinase inhibition resulted in significant neuroprotection in a majority of these models, it failed to restore function in some models (e.g., spinal cord injury). However, none of the previous studies carefully characterized or investigated p38α MAPK inhibitors in vivo for their independent effects on amyloid and tau pathologies, which are each relevant to AD and related non-AD tauopathies.

In the current study, we have utilized MW01-10-181SRM (or MW181), a highly selective p38α MAPK inhibitor [26], for its effects on p38α MAPK activity and tau phosphorylation in vitro and in 20-month-old hTau mice with advanced stages of disease. We report here that MW181 suppresses p38α MAPK activation, reduces tau phosphorylation, and decreases proinflammatory cytokine production. We also show that MW181 may prevent cognitive impairment in the aged hTau mice, possibly attributed to increasing synaptophysin levels. The data with MW181 were compared in several key experiments with results obtained using the mixed kinase inhibitor SB239063. In most experiments the results were congruent, allowing normalization of our results to those in the literature. The results with MW181, however, were remarkable in their further validation of p38α MAPK as a potential therapeutic target for tauopathies.

## Methods

### In-vitro experiments

#### Primary cortical neuronal cultures

**Fig. 1:**
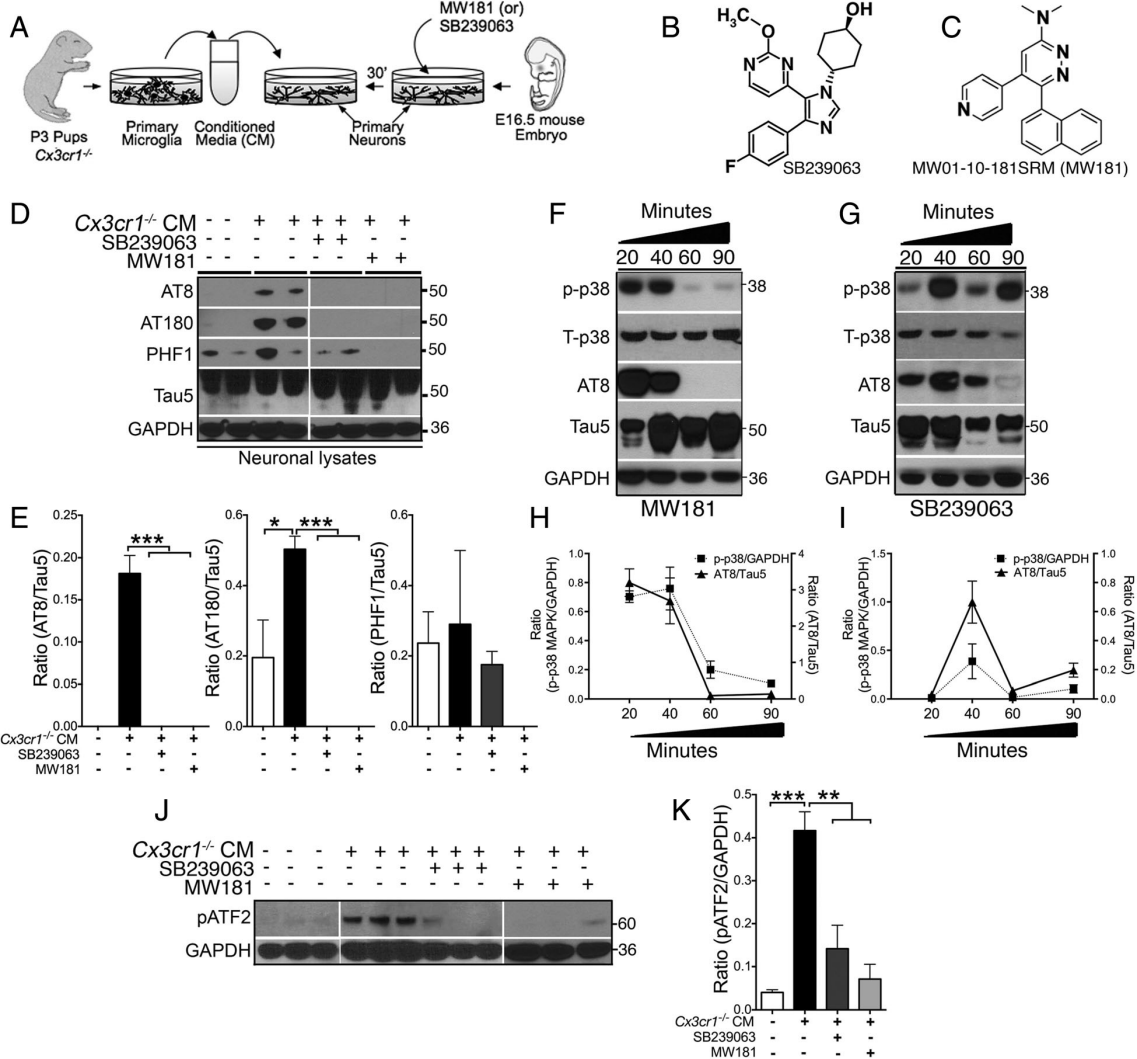
SB239063 and MW181 reduce inflammation-induced hyperphosphorylation of tau, p38α MAPK, and ATF2 activation in primary neurons. **a** Schematic showing 30-min pretreatment of 21 DIV *Cx3cr1* ^*+/+*^ primary cortical neurons with MW181 (2 μM), SB239063 (100 μM), or vehicle (Veh) followed by treatment with 25% *Cx3cr1* ^*–/–*^ microglial conditioned media (CM) for 90 min prior to biochemical analysis of neuronal lysates. **b**, **c** Structural formulae of SB239063 (adapted from [68]) and MW181 (adapted from [26]). **d**, **e** *Cx3cr1* ^*–/–*^ microglial CM significantly induced tau phosphorylation on AT8 and AT180 sites. Pretreatment of neurons with SB239063 or MW181 significantly reduced CM-induced tau phosphorylation on AT8 and AT180 sites. Quantifications are shown in **e** (*n* = 3 independent cultures, mean ± SEM of integrated density value (IDV) ratios as labeled. *p < 0.05; ***p < 0.001; one-way ANOVA with Tukey’s multiple comparison test). **f**–**i** MW181 (**f**) or SB239063 (**g**) neuronal treatment showing reduction in the activated p-p38α MAPK (pT180/pY182) and AT8 site tau phosphorylation with a maximum reduction at 60 and 90 min time points (for MW181), and at 20 and 60 min time points (for SB239063) post CM treatment, which was considered as 0 min. Quantifications are shown in **h** (for MW181) and **i** (for SB239063). *N* = 3 independent cultures, mean ± SEM of IDV ratios as labeled. **j**, **k** Elevated levels of phosphorylated ATF2 (pT71) at 90 min after *Cx3cr1* ^*–/–*^ microglial CM treatment. The pATF2 level was reduced by 30-min pretreatment with SB239063 and MW181. Quantifications are shown in **k** (*n* = 3 independent cultures, mean ± SEM of IDV ratios of pATF2/GAPDH; **p < 0.01; ***p < 0.001; one-way ANOVA with Tukey’s multiple comparison test)

Neuronal cultures were prepared from embryonic day 16.5 (E16.5) C57BL/6 J mouse embryos as described previously [41]. Primary cortical neurons were seeded onto poly-l-lysine coated six-well plates at a density of 0.5 × 10^6^ cells/well for conditioned media (CM) experiments (Fig. 1a). Cultures were grown for 21 days in vitro (DIV) at 37 °C in neurobasal media with B-27 supplement in humidified 5% CO_2_/95% air prior to any treatment.

#### Primary microglial culture

Microglial cultures were prepared from postnatal day 3 (P3) pups from *Cx3cr1* ^*–/–*^ mice litters [42] as described previously [43]. Briefly, mixed glial cells were first cultured and grown in a T-75 cm^2^ flask seeded at a density of 1.0 × 10^5^–1.2 × 10^5^ cells/cm^2^ in 10% fetal bovine serum/Dulbecco’s modified eagle medium (FBS/DMEM F12 or complete growth media). After 14 DIV, a differential trypsinization [43] protocol was utilized to remove the astrocytes in the flasks and the pure population of microglia was seeded at a density of 0.25 × 10^6^ cells/well in a six-well plate (Fig. 1a) in 2% FBS/DMEM to ensure adherence. Next, the complete growth media were replaced with neurobasal media (with no B27 supplement) 24 h prior to the co-culture experiment to match the culture media of primary neurons for CM studies (see later).

### Neuron-microglia CM experiments and pharmacokinetics

Primary neuronal and microglial cultures were prepared as already described. 21 DIV primary cortical neurons were pretreated for 30 min with  p38α MAPK inhibitors (SB239063, 100 μM (catalog number S0569; Sigma) dissolved in DMSO; or MW181, 2 μM dissolved in saline—0.9% NaCl/H_2_O, pH 7.4) or VEH (saline). After 30 min, 25% of the media was removed from each well with primary neurons and was replaced with *Cx3cr1* ^*–/–*^ microglia CM (harvested just before adding to the neuronal wells without any prior centrifugations). After 90 min, neurons were lysed in 1× lithium dodecyl sulfate (LDS) sample buffer with sample reducing agent (RA) buffer (a total volume of 100 μl per two wells in a six-well plate) and sonicated for 30 seconds. For the time-course experiments, neurons were first pretreated with the p38α MAPK inhibitors (SB239063 at 100 μM final concentration or MW181 at 2 μM final concentration) or vehicle (saline) 30 min prior to the addition of *Cx3cr1* ^*–/–*^ microglia CM. We chose 2 μM for MW181 based on our previous studies where a dose of 5 μM showed significantly reduced levels of IL-1β by LPS-stimulated BV2 cells [26]. Similarly, 100 μM of SB239063 was selected based on a previous study where 84% downregulation of IL-1β mRNA was observed in microglial cells in an organotypic hippocampal slice culture model [44]. At 20, 40, 60, and 90 min after the addition of the *Cx3cr1* ^*–/–*^ microglia CM, the neuronal lysates were prepared as already described. All experiments were performed in triplicate with independent cultures.

### In-vivo experiments

#### Mice

The hTau [45] (expressing human *MAPT* and deficient for endogenous mouse *Mapt*) mice were bred and maintained in our colony. MK2^–/–^ mice [46] were a kind gift from Dr Matthias Gaestel, Medical School Hannover, provided by Dr Ellen Beswick at the University of New Mexico. Both hTau and MK2^–/–^ mice were in the C57BL/6 J background. We utilized mixed genders of these mice in the present study (Table 1). All experimental protocols involving animals were performed in accordance with the US National Institutes of Health guidelines on animal care and were approved by the Institutional Animal Care and Use Committees of both the University of New Mexico and the Cleveland Clinic Foundation.

**Table 1:** Genotype, group size, gender, and age of the mice utilized in the current study

Treatment group	Group size (*n*)	Gender
20-month hTau + Veh	12	10 female
		2 male
20-month hTau + SB239063	4	4 female
20-month hTau + MW181	8	6 female
		2 male
6-month hTau + Veh	3	1 male
		2 female
6-month hTau + MW181	4	1 male
		3 female
6-month C57BL/6 J (non-Tg)	3	3 male
2-month-old MK2^–/–^ + Veh	3	3 male
2-month-old MK2^–/–^ + LPS	4	4 male
2-month-old C57BL/6 J (non-Tg) + Veh	3	3 male
2-month-old C57BL/6 J (non-Tg) + LPS	4	4 male

*LPS* lipopolysaccharide, *Tg* transgenic, *Veh* vehicle

### Oral Gavage (p.o.) experiments

The hTau mice (20 months of age) were divided into three groups and were administered: SB239063 (in 0.1% dimethyl sulfoxide (DMSO) in H_2_O, with 50% polyethylene glycol (PEG) 400 and carboxymethyl cellulose as described previously [47] and at a final concentration of 5 mg/kg, body weight (b.w.)); MW181 (dissolved in 0.9% saline; administered at a final concentration of 1 mg/kg, b.w.); or saline (vehicle) orally using an 18-gauge stainless steel oral gavage needle (catalog number FNS-18-2; Kent Scientific) daily, over the course of 14 days. All animals receiving treatment were monitored for weight loss/gain, grooming changes and posture over 14 days; no differences were noted.  We selected 1 mg/kg MW181 based on our previous study, where 5 mg/kg b.w. of MW181 was able to attenuate Aβ-induced synaptic and cognitive dysfunction in a mouse model of AD [26]. Following the treatment, animals were sacrificed (described later).

### LPS injections

Lipopolysaccharide (LPS, catalog number L2630; Sigma) was obtained from phenol-extracted *E. coli* and the lyophilized powder was dissolved in Hanks’ balanced salt solution (HBSS, catalog number H9269; Sigma) at a stock concentration of 1 mg/ml. Nontransgenic and MK2^–/–^ mice were treated with a single dose of LPS (10 mg/kg, b.w., intraperitoneally (i.p.)). Animals were sacrificed 24 h post injection as described later.

### Antibodies and reagents

#### MAPT antibodies

The following antibodies against tau were used: AT8 (pS199/pS202), AT180 (pT231), and Tau5 (Thermo Fisher Scientific) and PHF-1 (pS396/pS404; provided by Peter Davies, Albert Einstein College of Medicine) were utilized. Phosphorylated p38 MAPK (pT180/pY182), phosphorylated ATF2 (pT71), and phosphorylated MK2 (pT233) antibodies were from Cell Signaling and total p38α MAPK antibody was from Thermo Fisher Scientific. The synaptophysin antibody was a kind gift from Dr Michael Wilson (deceased), and GAPDH antibody was purchased from Millipore. The following antibodies were used to mark immune cells: B-cell specific antibody B220-biotin/CD45R-biotin (R&D Systems), T-cell specific antibody CD3 (R&D Systems), microglia/macrophage specific antibodies Iba1 (Wako), and CX3CR1 (R&D Systems) were utilized.

### Tissue preparation and measurement of hippocampal wet weight

The mice were anaesthetized and transcardially perfused with 0.125 M phosphate buffer (PB). Following perfusion, the brains were removed, the left hemisphere was immersion fixed in 4% paraformaldehyde in PB (4% PFA/PB), the right hemisphere was microdissected into the cortex and hippocampus, wet weights were recorded, and the tissues were snap frozen in liquid nitrogen for subsequent biochemical analysis. The rest of the right hemispheres were weighed and snap frozen in liquid nitrogen for subsequent mRNA extraction.

### SDS-PAGE and western immunoblotting

Proteins were homogenized in 10% weight/volume Tissue Protein Extraction Reagent (T-PER®; Thermo Fisher Scientific) and soluble hippocampal lysates were resolved via SDS-PAGE and immunoblotted as described previously [22]. The dilutions of primary antibodies utilized were as follows: AT180, 1:2500; pATF2, pMK-2, AT8, PHF-1, and synaptophysin, 1:5000; GAPDH and Tau5, 1:10,000; and phospho-p38α MAPK and total p38α MAPK, 1:1000.

### Sarkosyl-insoluble assay

The Sarkosyl-insoluble fraction of MAPT was isolated from hippocampal tissues as described before [48], with minor modifications described previously [22].

### Gene expression analysis

RNA from the brain was extracted using the TRIzol® reagent as described by the manufacturer (Thermo Fisher Scientific). Total RNA (100 ng/μl) was converted to cDNA using the High Capacity cDNA Reverse Transcription kit and amplified using specific TaqMan probes (gene expression markers: interferon gamma (IFN-γ, Mm00801778_m1), IL-1β (IL-1β, Mm00434228_m1), tumor necrosis factor alpha (TNFα, Mm00443258_m1), interleukin-6 (IL-6, Mm00446191_m1), interleukin-4 (IL-4, Mm00445259_m1), Chitinase 3-like 3 (YM1, Mm00657889_m1), Arginase 1 (ARG1, Mm00475988_m1), and MAPAPK (MK2, Mm01288465_m1)) and GAPDH was used as a house keeping gene for normalization. The qPCR assays were run on the StepOnePlus® Real-Time PCR System (all reagents were purchased from Thermo Fisher Scientific).

### Immunofluorescence, immunohistochemistry, and quantitative morphometry

Free-floating sections (30 μm thick) were processed for immunofluorescence or immunohistochemical analysis as described previously [22]. Briefly, sections were first incubated in 10 mM sodium citrate buffer (pH 6.0) for 10 min at 95 °C for antigen retrieval, washed in PBS with 0.1% Tween (PBST), and quenched with 0.3% H_2_O_2_ in PBST for 15 min (only for immunohistochemistry). Sections were blocked for 1 h at room temperature with the 5% normal sera (goat/donkey, from the animal species in which the secondary antibodies were raised). The sections were incubated with primary antibodies at the following dilutions: Iba1 and B220-biotin, 1:500; CD3, 1:50; CX3CR1, 1:100; and AT180, 1:250. After washing in PBST, the sections were incubated with Alex-Flour® conjugated secondary antibodies (1:1000 for immunofluorescence; ThermoFisher Scientific) or biotin (1:250 for immunohistochemistry; Vector Laboratories). Sections were then either mounted in DAPI Hardset Reagent (for immunofluorescence) or incubated with Avidin:Biotinylated enzyme Complex (ABC reagent, for immunohistochemistry; Vector Laboratories) reagent for 1 h at RT. The immunoreactive signals were revealed by developing sections in SigmaFast® 3,3′-diaminobenzidine (DAB) tablets (Sigma-Aldrich). Bright-field and epifluorescence images were acquired using a Leica DMR upright fluorescence/bright-field microscope.

Tiff images were first converted from 24-bit RGB to 8-bit gray scale using NIH Image J software for quantitative morphometric analysis (AT180, Iba1, CD3, and B220). Next, the threshold was set using the *image > Adjust > Threshold* tool for individual images. The threshold in the immunostained areas was set with the *Huang* auto-threshold method (display mode black and white) with minimum and maximum threshold values of 0 and 90 respectively. These values were inversed for the immunofluorescence images. After applying the threshold mask, the total immunoreactive area was calculated using the *Analyze > Measure* tool. Prior to running the *measurement* tool, the measurements were limited to the highlighted area threshold by using the tool *Analyze > Set Measurement* and selecting the “Limit to threshold” option. Three different fields (covering the dorsolateral hippocampal area) per section and three random sections per mouse brain were scored by an individual blinded to the treatment/genotype. For the quantification of CX3CR1^+^ cells, three random sections per mouse brain (*n* = 3 per condition) were scored manually for the amount of CX3CR1^+^ microglia that appeared activated (with swollen cell body compared with normal appearing microglia and shorter processes) in the CA1 region of the hippocampus. Mean ± SEM values of the CX3CR1^+^ microglia were plotted. Slides were coded prior to scoring and the analysis was performed in a blinded manner.

### Y-maze behavioral analysis

A symmetrical Y maze was used to evaluate spatial working memory [49]. Mice were placed in the center of the Y maze and allowed to freely explore for one 5-min trial. The sequence of arm entries was recorded and analyzed for spontaneous alternation behavior. One spontaneous alternation was documented when a mouse consecutively entered three different arms. Percent spontaneous alternation was calculated by dividing the number of alternations by the maximum number of alternation opportunities minus two. The average number of arms entered over a 5-min period was plotted.

### Statistical analysis

Unless otherwise indicated, comparisons between the two groups were carried out via unpaired *t* test; comparisons between multiple treatment groups were performed via one-way or two-way analysis of variance (ANOVA) with indicated multiple comparison post-hoc tests. All statistical analyses were performed using GraphPad Prism®.

## Results

### MW181 and SB239063 treatment rescued inflammation-induced phosphorylation of tau, p38α MAPK, and ATF2 in primary neurons

To assess the effects of two brain-permeable p38α MAPK inhibitors (MW181 and SB239063) on the inflammation-induced tau hyperphosphorylation, we pretreated primary cortical neurons either with vehicle (VEH/saline), SB239063, or MW181 for 30 min and then stimulated the primary neurons with CM derived from *Cx3cr1* ^*–/–*^ primary microglia (Fig. 1a–c). After 90 min of CM treatment, neurons were lysed and the lysates were processed to detect the level of tau phosphorylation. As reported previously [22], exchange of 25% of the neuronal supernatant with *Cx3cr1* ^*–/–*^ microglia CM led to an increase in tau phosphorylation on S199/S202 (AT8) and T231 (AT180) sites (Fig. 1d, e). Notably, the S396/S404 (PHF1) site also showed a small increase in tau phosphorylation; however, because of high variability and the fact that mouse tau has been known to display higher basal levels of phosphorylation on the PHF1 site, CM treatment did not induce significant phosphorylation on the PHF1 site (Fig. 1d, e). Importantly, preincubation of neurons with MW181 or SB239063 completely blocked AT8 site and AT180 site tau phosphorylation (Fig. 1d, e). Interestingly, PHF1 site phosphorylation of tau was also completely abolished with MW181 pretreatment (Fig. 1d, e).

To assess the time course of the effects of both MW181 and SB239063, we first pretreated neurons with MW181 or SB239063 for 30 min, and then added the *Cx3cr1* ^*–/–*^ CM. 20, 40, 60, and 90 min after the addition of CM, and neurons were harvested for the western blot analysis. MW181 pretreatment showed its maximum effect in reducing phosphorylation of p38α MAPK (T180/Y182) and tau (AT8 site) at 60 and 90 min (Fig. 1f, h). Whereas the phosphorylation of tau would reflect a potential direct kinase-substrate event, the phosphorylation of p38α MAPK by a validated active-site binding, kinome-selective probe like MW181 [26] would reflect an indirect effect mediated by feedback mechanisms. In order to confirm further that the effects of MW181 and SB239063 were via a p38α MAPK-mediated pathway, we examined the T71 phosphorylation of ATF2, a well-established substrate of p38α MAPK [50]. Incubation of primary neurons with *Cx3cr1* ^*–/–*^ CM for 90 min increased phosphorylation of pATF2 on T71, which was significantly blocked by a 30-min pretreatment with MW181 (Fig. 1j, k). In contrast to MW181, SB239063 showed a biphasic effect with an initial drop in the p-p38α MAPK and AT8 site tau phosphorylation at 20 min and then again at 60 min after *Cx3cr1* ^*–/–*^ CM treatment (Fig. 1g, i). There was a sharp spike in both p38α MAPK and AT8 site tau phosphorylation at the 40-min time point (Fig. 1g, i). The time-dependent variability of SB239063 effects on a phosphorylation cascade in a cell culture system might reflect its multi-kinase inhibitor activity (e.g., [34, 51]), where each kinase target may have a role that varies quantitatively during the phenotypic change. This was not pursued further as part of this investigation. Regardless, SB239063 also blocked the increased phosphorylation of pATF2 on T71 (Fig. 1j, k).

### Oral administration of MW181 reduces tau phosphorylation and aggregation and improves working memory in 20-month-old hTau mice with an advanced stage of tau pathology

**Fig. 2:**
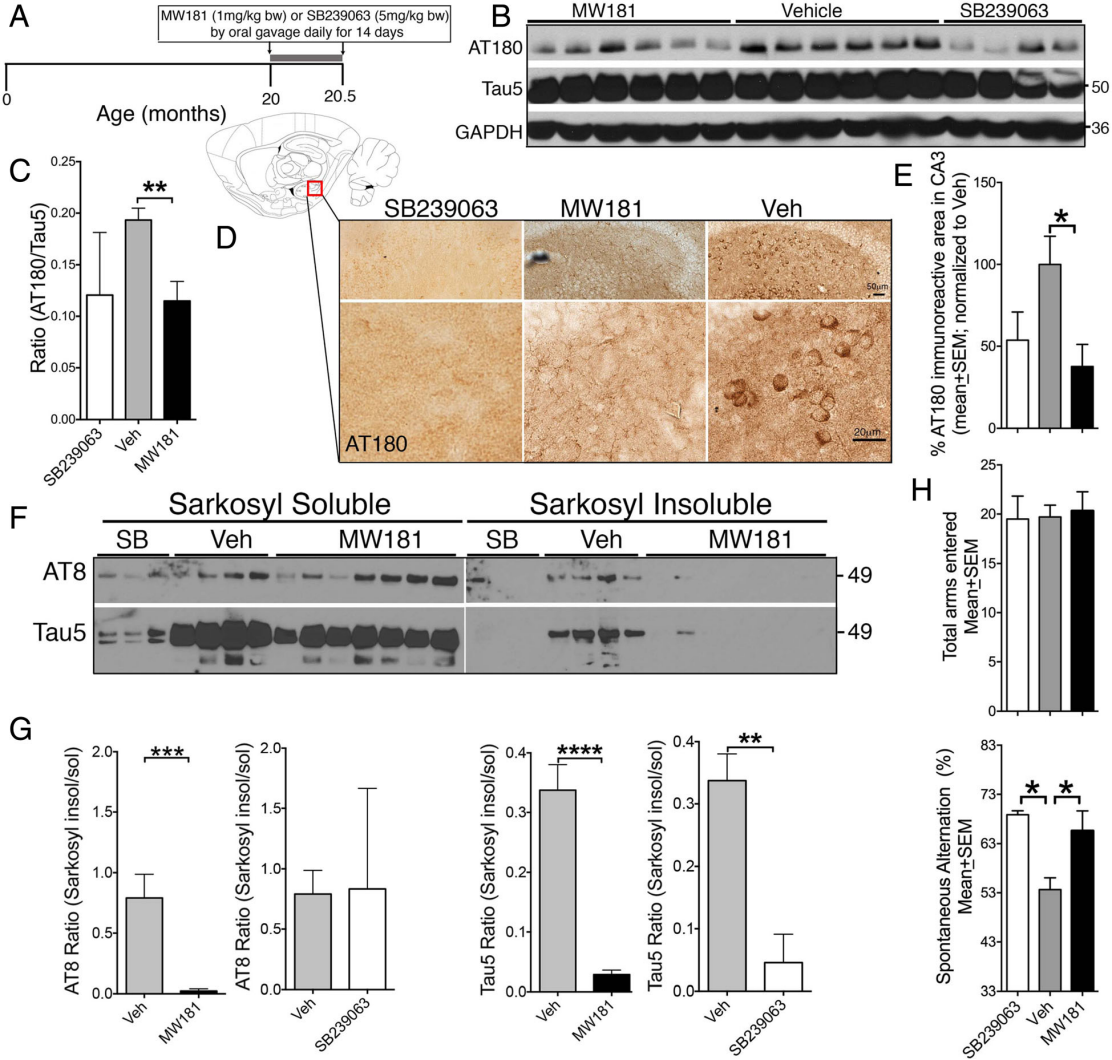
Oral administration of MW181 reduces hyperphosphorylated and aggregated tau in 20-month-old hTau transgenic mice. **a** Treatment schema for intervention for MW181 (1 mg/kg b.w.), SB239063 (5 mg/kg b.w.), or vehicle (saline) administration via oral gavage once daily for a total of 14 days. After the end date (age 20.5 months), mice were sacrificed for biochemical and neuropathological analysis. **b** Western blots of detergent soluble hippocampal lysates from 20.5-month-old hTau mice treated with MW181 and SB239063 show significant reduction of AT180 in MW181-treated mice compared with the vehicle-treated group. **c** Quantifications of AT180/Tau5 ratio show statistically significant (*n* = 4 for SB239063, *n* = 6 for MW181, and *n* = 6 for vehicle; mean ± SEM of IDV ratios; **p < 0.01; one-way ANOVA with Tukey’s multiple comparison test) reduction in the AT180/Tau5 ratio in the MW181-treated compared with vehicle-treated hTau mice. **d** Outline of the sagittal brain section identifying the hippocampal region used for quantitative morphometry for AT180. Immunohistochemistry revealed reduced AT180 specifically in the CA3/dentate gyrus of hippocampus of SB239063-treated and MW181-treated hTau mice compared with vehicle-treated controls. *Scale bar*: 50 μm (*top panels*) and 20 μm (*bottom panels*). **e** Quantitative morphometry for AT180 for groups in **d** (*n* = 3 mice per genotype; two random fields in three sections per brain were quantified; percentage mean ± SEM of AT180 immunoreactive area in the CA3 region of hippocampus; normalized to vehicle-treated group, which was 100%; *p < 0.05; one-way ANOVA with Tukey multiple comparison test). **f, g** Reduced levels of Sarkosyl-insoluble and AT8^+^ tau in the hippocampi of MW181-treated but not with SB239063-treated 20-month-old hTau mice compared with age-matched, vehicle-treated controls. **g** Quantifications of Sarkosyl-insoluble/Sarkosyl-soluble ratios for AT8 (*left graphs*) and Tau5 (*right graphs*) show statistically significant (*n* = 5 for Veh, *n* = 7 for MW181, and *n* = 3 for SB239063; mean ± SEM of IDV; **p < 0.01, ***p < 0.001, **** *p* <0.0001; unpaired *t* test) reduction in Tau5 in the Sarkosyl-insoluble fraction of MW181-treated and SB239063-treated hTau mice. **h** Twenty-month-old hTau mice treated with vehicle exhibited significantly reduced (*p < 0.05; one-way ANOVA with Tukey multiple comparison test; *n* = 5 for vehicle, *n* = 4 for SB239063, and *n* = 6 for MW181) percentage of spontaneous alternation compared with MW181-treated hTau mice in the Y-maze test. No differences in the total number of arms entered were noted. *Veh* vehicle

To test the in-vivo efficacy of MW181 and SB239063, three separate cohorts of 20-month-old hTau mice were treated with MW181 (1 mg/kg b.w.; p.o.), SB239063 (5 mg/kg b.w.; p.o.), or vehicle (physiological saline) once daily for 14 days via oral gavage (Fig. 2a). Twenty-four hours after the last dose, mice were sacrificed and the hippocampi were processed for western blot analysis. A modest reduction in the level of AT180 site tau phosphorylation was observed in SB239063-treated hTau mice compared with the vehicle-treated group (Fig. 2b). Strikingly, once-daily dose of MW181 showed a statistically significant reduction in the AT180 site tau phosphorylation following 14 days of treatment in the aged hTau mice (Fig. 2b, c).

To confirm the reduction of AT180 site tau phosphorylation, we performed immunohistochemistry on brains from 20-month-old vehicle-treated, SB239063-treated, or MW181-treated hTau mice. We noted a significant difference in the number of AT180^+^ neurons in the basolateral hippocampus, specifically encompassing the CA3 and dentate gyrus (DG) regions (Fig. 2d), but not in other regions of the hippocampus. Interestingly, quantitative morphometric scoring of the percentage of AT180^+^ area in two different fields covering CA3/DG with three sections per mouse and from three mice per group showed a statistically significant decrease in AT180^+^ neurons in MW181-treated but not in SB239063-treated hTau mice (Fig. 2e).

Next, we performed the Sarkosyl-insoluble assay in the hippocampus to determine whether the reduction in AT180 site tau hyperphosphorylation in the MW181-treated and SB239063-treated group led to any alterations in tau aggregation. Notably, all four vehicle-treated 20-month-old hTau mice showed a very clear presence of Tau5^+^ bands (total tau) in the Sarkosyl-insoluble fraction compared with MW181-treated and SB239063-treated hTau mice (Fig. 2f), suggesting this treatment reduced tau aggregation/NFT pathology. Sarkosyl-insoluble tau in the vehicle-treated group was phosphorylated at the AT8 site (Fig. 2f). Analysis of the Sarkosyl-insoluble/Sarkosyl-soluble ratio for Tau5 showed a statistically significant reduction in the levels of Sarkosyl-insoluble aggregated tau in MW181-treated and SB239063-treated hTau mice compared with the vehicle-treated group (Fig 2g). Because of the variability, the difference in the Sarkosyl insoluble/soluble ratios for AT8 showed statistically significant reduction only with MW181, but not with SB239063 treatment, compared with vehicle (Fig. 2g).

Previous studies have suggested that hTau mice exhibit various behavioral and cognitive impairments by 20 months of age [52]. To determine whether MW181-mediated reduction in tau pathology translates to improved behavioral function, 20-month-old hTau mice treated with vehicle, SB239063, or MW181 were subjected to Y-maze behavioral analysis. While the total number of arm entry was indistinguishable between vehicle-treated, SB239063-treated, and MW181-treated groups (Fig. 2h), hippocampal-dependent working memory was significantly improved in SB239063-treated and MW181-treated hTau mice compared with vehicle-treated controls because the SB239063-treated or MW181-treated hTau mice displayed a significantly elevated spontaneous alternation score in the Y-maze test (Fig. 2h). Together, these results suggest that SB239063 and MW181 could reduce tau hyperphosphorylation and aggregation as well as improve working memory in 20-month-old hTau mice.

### In-vivo administration of MW181 reduces the level of pMK2 and pATF2, and increases synaptophysin in 20-month-old hTau mice

**Fig. 3:**
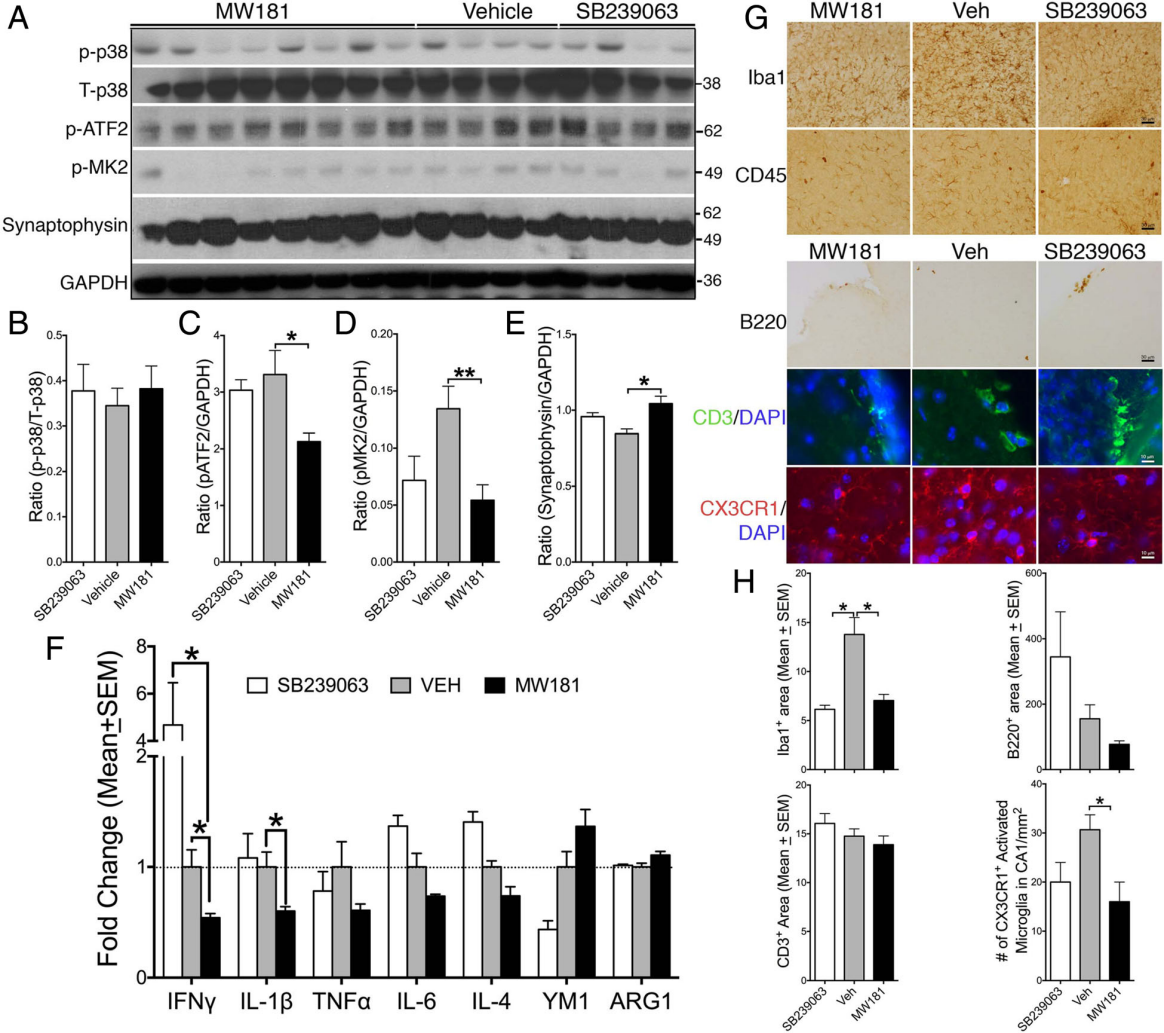
MW181 reduced the levels of pATF2 and pMK2, increased levels of synaptophysin, and blunted the expression of IFNγ and IL-1β in 20-month-old hTau mice. **a** Western blot analysis of hippocampal lysates shows no significant difference in p-p38α MAPK (pT180/pY182) levels, but reduced levels of phosphorylated ATF2 (pT71) and phosphorylated MK2 (pT334) and increased levels of synaptophysin were observed with MW181 treatment. **b**–**e** Quantification shows no difference for p-p38α MAPK, but statistically significant reduction (*n* = 3 for SB239063, *n* = 8 for MW181, and *n* = 5 for vehicle; mean ± SEM of IDV ratios; *p < 0.05; **p < 0.01; one-way ANOVA with Tukey’s multiple comparison test) in the pATF2/GAPDH and pMK2/GAPDH ratios in MW181-treated hTau mice compared with vehicle-treated hTau mice. None of these proteins were seen to be statistically altered with SB239063 treatment. **f** Quantitative real-time PCR analysis for various markers of classical activation or M1 state (IFNγ, IL-1β, TNFα, and IL-6) and alternative activation or M2 state (IL-4, YM1, and ARG1) shows a significant reduction (*n* = 3 for SB239063 and *n* = 6 for MW181 and vehicle; mean ± SEM of IDV ratios; *p < 0.05; two-way ANOVA with Tukey’s multiple comparison test) in the levels of IFNγ and IL-1β in the MW181-treated hTau mice compared with vehicle-treated hTau mice. **g** Immunohistochemical and immunofluorescence analysis shows Iba1^+^ microglia and CD45^+^ microglia/macrophages in the CA3 region of hippocampus, B220^+^ B cells and CD3^+^ T cells in the meninges, and CX3CR1^+^ microglia in the CA3 region of the hippocampus in MW181-treated, Veh-treated, or SB239063-treated 20-month-old hTau mice. *Scale bars*: 30 μm for Iba1, CD45, and B220; 10 μm for B220 and CD3. **h** Quantitative morphometry for Iba1, B220, CD3, and CX3CR1 for immunochemistry in **g** (*n* = 3–4 mice per genotype); four random fields in three sections per brain were quantified; mean area (± SEM) immunoreactive for Iba1, B220, and CD3 in the hippocampus (for Iba1) or in the meninges (for CD3 and B220). For CX3CR1, the number of CX3CR1^+^ microglia displaying activated morphology (reduced processes and swollen cell body) was counted manually in the CA1 region of hippocampus and expressed as number of cells per mm^2^ (*p < 0.05; one-way ANOVA followed by Tukey multiple comparison post-hoc test). Note that B220 and CD3 showed a reduced trend in MW181-treated groups but were not statistically significant, unlike Iba1 and CX3CR1 which were significant. *Veh* vehicle

To better link the reduced tau hyperphosphorylation and aggregation in the MW181-treated aged hTau mice to a decrease in p38α MAPK activity, we assessed the phosphorylation of downstream pathway targets: pATF2 (T71) and pMK2 (pT334). Unlike cell culture studies, the levels of p-p38/T-p38α MAPK in MW181-treated or SB239063-treated mice did not significantly decrease compared with vehicle-treated groups (Fig. 3a, b). MW181-treated aged hTau mice showed a significant reduction in both pATF2 and pMK2 compared with vehicle-treated groups (Fig. 3a, c, d). These results indicate that MW181 can significantly affect the in-vivo catalytic activity of p38α MAPK with a resultant reduction in the site-specific phosphorylation of known substrates (T71 and T334 sites in ATF2 and MK2, respectively). This was not observed with SB239063.

A previous study demonstrated elevated synaptophysin levels following p38α MAPK inhibition using a mixed kinase inhibitor on IL-1β stressed neurons in culture [24]. To determine whether in-vivo treatment with MW181 alters synaptophysin levels, we performed western blot analysis for synaptophysin in the hippocampus of MW181-treated 20-month-old hTau mice. There was a statistically significant increase in synaptophysin in MW181-treated 20-month-old hTau mice compared with vehicle-treated mice (Fig. 3a, e). SB239063 induced a modest increase in synaptophysin levels; however, the difference (versus vehicle control) was not statistically significant when analyzed with multiple comparisons via one-way ANOVA (Fig. 3a, e). It is important to note that effect of SB239063 on pMK2 and synaptophysin levels was arithmetically superimposable with those of MW181, although statistical significance was not reached because of the limited number of animals used in SB239063-treated groups compared with those in MW181-treated groups.

### MW181 blocks IFNγ and IL-1β expression and promotes alternative immune activation

Activation of both ATF2 and MK2 may lead to the expression of various proinflammatory cytokines/chemokines including IL-1β. Inhibition of p38α MAPK by MW181, but not by SB239063, led to a significant decrease in both IFNγ and IL-1β expression in the 20-month-old hTau mice (Fig. 3f). Interestingly, both IL-6 and TNFα levels also displayed marginal reduction with MW181 treatment during this same time. However, the differences were not statistically significant (Fig. 3f). Furthermore, an increase in the level of YM1 and ARG1, which are markers of alternative activation, showed an increased trend in MW181-treated hTau mice, but did not reach statistical significance (Fig. 3f). To determine whether the pattern of gene expression observed for the inflammatory cytokines/chemokines would reflect the morphological alterations of microglia and peripherally derived macrophages, we performed a series of immunohistochemical and immunofluorescence experiments. Notably, Iba1^+^/CX3CR1^+^ microglia appeared more resting in the CA3 region of hippocampus of MW181-treated and SB239063-treated 20-month-old hTau mice compared with vehicle-treated hTau mice (Fig. 3g). Qualitatively, no differences were observed for CD45^+^ microglia/peripherally derived macrophages across different treatment groups (Fig. 3g), and therefore no further quantitative morphometric analysis was performed. Since IFNγ is primarily secreted by T cells and was more than four-fold higher in SB239063-treated hTau mice compared with vehicle-treated controls and that MW181 treatment significantly reduced IFNγ expression levels, we also assessed the presence of T cells and B cells in brain sections of hTau mice treated with vehicle, MW181, or SB239063. Immunofluorescence analysis revealed the presence of qualitatively more B220^+^ B cells and CD3^+^ T cells in the meninges of vehicle-treated and SB239063-treated 20-month-old hTau mice compared with those treated with MW181 (Fig. 3g). We did not observe presence of any parenchymal B cells/T cells in any treatment groups. Upon quantification, we observed statistically significant reduction in the Iba1 immunoreactivity and the number of CX3CR1^+^ activated microglial cells, but not in the CD3^+^ T cells/B220^+^ B cells, in MW181-treated or SB239063-treated groups compared with vehicle-treated 20-month-old hTau mice (Fig. 3h). Together, these results suggest that primarily MW181, but to a lesser extent SB239063, are both capable of suppressing proinflammatory responses, while inducing alternative, more neuroprotective, anti-inflammatory responses in the brain.

### MK2^–/–^ mice are resistant to LPS-induced tau phosphorylation and show reduced levels of total p38α MAPK

**Fig. 4:**
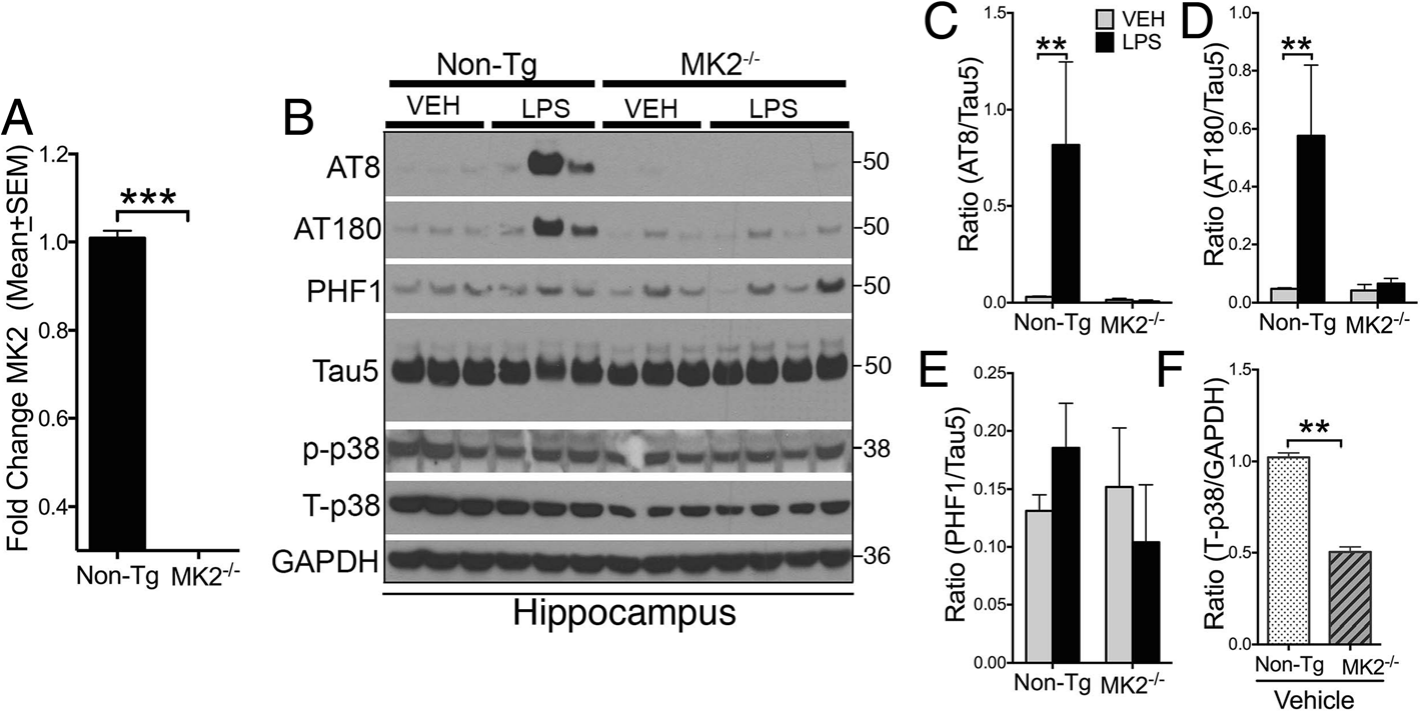
MK2-deficient mice are resistant to LPS-induced tau phosphorylation and show reduced p38α MAPK. **a** Quantitative real-time PCR analysis for MK2 in the brains of 2-month-old nontransgenic vs. MK2–/– mice confirms a significant knockdown of MK2 in these mice (*n* = 4 for non-Tg and *n* = 3 for MK2–/– mice; ***p < 0.0001; unpaired t test; mean ± SEM). **b** Western blots of detergent soluble hippocampal lysates from 2-month-old nontransgenic and MK2^–/–^ mice treated with either vehicle (*VEH*) or LPS (10 mg/kg b.w.; intraperitoneal; single dose for 24 h). Note the LPS-induced AT8 and AT180 site tau phosphorylation in LPS-treated nontransgenic mice, which is reduced in MK2^–/–^ mice. No significant difference is seen in PHF1-antibody specific tau phosphorylation and phosphorylated p38α MAPK (pT180/pY182) levels. However, the total p38α MAPK levels are reduced in MK2^–/–^ mice. **c–e** Quantifications of the western blots in **b** show a statistically (*n* = 3 mice per group; mean ± SEM of IDV ratios of AT8/Tau5 and AT180/Tau5; **p < 0.01; two-way ANOVA with Bonferroni’s multiple comparison test) significant increase in IDV ratios for AT8/Tau5 and AT180/Tau5 in LPS-treated non-Tg, but not in LPS-treated MK2^–/–^ mice. **f** Quantifications of the total-p38α MAPK levels show statistically significant reduction in the levels of total-p38α MAPK/GAPDH IDV ratio (*n* = 3 per group; mean ± SEM; **p < 0.01; unpaired *t* test) in MK2^–/–^ mice compared with nontransgenic mice. *Tg* transgenic

We have demonstrated previously that intraperitoneal administration of LPS results in elevated tau phosphorylation within the brain and that mice deficient for toll-like receptor 4 (TLR4) or IL-1β receptor (IL-1R) are resistant to this LPS-induced tau phosphorylation [22]. Since p38α MAPK inhibition by MW181 leads to both MK2 deactivation and reduced tau phosphorylation (Fig. 3), we tested whether MK2 deficiency would prevent LPS-induced tau phosphorylation in vivo. Two-month-old nontransgenic and MK2^–/–^ mice were administered a single dose of LPS (10 mg/kg b.w.; i.p.). After 24 h, the hippocampi were processed for biochemical analysis. First, we assessed whether MK2^–/–^ mice are deficient for MK2 via quantitative real-time PCR analysis. Compared with 2-month-old nontransgenic mice, MK2^–/–^ mice displayed a statistically significant reduction in MK2 gene expression (Fig. 4a), suggesting the deficiency of MK2 in MK2^–/–^ mice. Next, we performed western blot analysis of the hippocampus to assess tau phosphorylation and p38 MAPK activation (phosphorylation). As reported previously [22], a single injection of LPS led to AT8 and AT180 site tau phosphorylation in nontransgenic mice (Fig. 4b–d). Unlike nontransgenic mice, MK2^–/–^ mice did not show an elevation in AT8 or AT180 site tau phosphorylation (Fig. 4b–d). There was also no significant effect of either LPS or MK2 deficiency on PHF1 site tau phosphorylation (Fig. 4b, e). Although there was no difference in the p-p38α MAPK (T180/Y182) levels observed in either nontransgenic or MK2^–/–^ groups, interestingly, total p38α MAPK levels were significantly reduced in vehicle-treated MK2^–/–^ mice compared with vehicle-treated nontransgenic mice (Fig. 4b, f). These results suggest that MK2^–/–^ mice are resistant to LPS-induced tau phosphorylation and that the deficiency of MK2 is correlated with reduced total-p38α MAPK levels.

## Discussion

There are three major findings in this study; MW181 can reduce inflammation-induced p38α MAPK activation and tau phosphorylation in primary neurons in vitro; MW181 can block tau phosphorylation and aggregation in a 20-month-old hTau mouse model of tauopathy with an advanced stage of tau pathology; and evidence consistent with a p38 MAPK-mediated pathway playing an important role in tau pathology was provided by the resistance of MK2^–/–^ mice to inflammation-induced tau hyperphosphorylation. These results identify p38α MAPK as a druggable target that can block inflammation-induced tau pathology and improve cognitive function in tauopathies.

Mitogen-activated protein kinase (p38α MAPK is one of the members of this large family of kinases) is associated with various cellular processes including cell division [53], immunity [54], differentiation, oncogenesis [55], neurogenesis, and apoptosis [56] as well as learning and memory [57] (reviewed in [58, 59]). In AD/tauopathies, MAPKs play various roles at different stages of disease [60]. p38α MAPK can play an important role both in amyloid-mediated and tau-mediated toxicities. First, p38α MAPK has been shown to mediate Aβ-induced expression of various proinflammatory cytokines/chemokines from microglial cells that causes neuroinflammation [25, 26, 29, 35]. Targeted inactivation of p38α MAPK with small molecule inhibitors prevents the production of proinflammatory cytokines, reduces neurodegeneration, and improves neuronal and cognitive functions in rodent models of amyloid pathology [25, 26, 29, 35]. Second, in neurons, p38α MAPK has been shown to act as an important kinase in inducing IL-1β-mediated hyperphosphorylation of tau [24, 61]. Our previous study suggested that enhancement of microglia-specific neuroinflammation via genetic deficiency of *Cx3cr1* resulted in accelerated tau pathology and cognitive impairment in an hTau mouse model of tauopathy [22]. Furthermore, hyperphosphorylation of tau in this model was mediated by upregulation of p38α the MAPK pathway, but not GSK3β or CDK5 pathways [22]. In a recent study, we demonstrated that adoptive transfer of reactive microglia from hTau-*Cx3cr1* ^*–/–*^ mice led to induction of tau hyperphosphorylation and aggregation within the brains of recipient mice, which was also mediated via the IL-1β–p38α MAPK signaling pathway [23].

To our knowledge, this report provides the most conclusive demonstration that selective inhibition of p38α MAPK alone can significantly reduce Sarkosyl-insoluble NFTs, increase synaptophysin, and reduce the expression of proinflammatory cytokines in a pure tauopathy model with advanced stages of disease. Notably, original reports on hTau mice suggested formation of mature tangles and neurodegeneration by 20 months of age [45, 62, 63]. However, a recent study by our group has suggested that deficiency of microglial *Cx3cr1* can enhance neuroinflammation and lead to the formation of Sarkosyl-insoluble tau in hTau mice as early as 2 months of age [23]. The potency of MW181 to reduce NFTs and rescue cognitive function suggests the therapeutic potential of selectively targeting p38α MAPK to attenuate tau pathology and cognitive impairment. While 1 mg/kg b.w. of MW181 was the only tested dose in the present study, different dosing and durations of treatment are key considerations during medical chemistry optimization of an in-vivo molecule for pharmacological safety. Just as our tauopathy studies described here were being completed, a recent drug development report [25] described a MW181 analog optimized for such key drug development compliances. While the anti-NFT effects of MW181 (via p38α MAPK inhibition) on 20-month-old hTau mice observed here are very intriguing, our study has some limitations. First, as far as we know, this is the first study targeting p38α MAPK in a 20-month-old mouse model of tauopathy, which is equivalent to approximately 60 years of age in humans, and demonstrating beneficial effects. Therefore, it should be validated in a separate study by a different group. Second, we did not perform parallel comparisons with nontransgenic mice administered with MW181, which might be important to determine whether MW181 effects are specific for transgenic mice or whether they have an overall affect in a normal system. Finally, in view of the normal physiological functions of p38 MAPK within the CNS, it is important to note that MW181 did not completely abolish the levels of activated forms of p38α MAPK. This further supports the possibility of modulatory effects of MW181 in selectively ablating the excessively activated forms of p38α MAPK.

Another important consideration related to the selectivity of MW181 for the α isoform of p38 MAPK is that MW181 is a representative member of efficacious and CNS-penetrant small molecule p38α inhibitors that large-scale kinome screens show are selective for the p38 MAPK family, lack crossover to major G-protein-coupled receptor (GPCR) agonist or antagonist classes based on functional screens, exhibit low toxicity at high doses, and are efficacious in an AD amyloid-relevant and progressive brain injury models [25, 26]. The selectivity of MW181 action is further indicated by the failure to bring about its CNS-relevant pharmacodynamic effects in p38α MAPK inhibitor-resistant mice [26, 28]. Further, the high-resolution crystal structure of MW181 and other members of this class of highly selective, in-vivo efficacious p38 MAPK inhibitor probes reveals that the structural basis of this selectivity is their common chemical scaffold that exploits specific three-dimensional features found only in p38 MAPK [25, 26]. Taken in its entirety, an extensive body of evidence indicates that the pharmacodynamic effects of this class of novel, selective inhibitors of CNS p38α MAPK are via modulation of endogenous p38α MAPK. In our previous study, the X-ray diffraction data revealed that the MW181 presence in the active site of the p38α MAPK renders the kinase to become inactive [25, 26]. This is an important consideration because the MW181-mediated inhibition may be entirely independent of the phosphorylation status of p38α MAPK on T180/Y182 sites. In other words, even if the p38α MAPK show unchanged T180/Y182 phosphorylation, binding of MW181 at the active sites makes the kinase inactive. Therefore, it is important to assess the activation status of p38α MAPK via assessing the phosphorylation of its downstream targets such as ATF2 and MK2. Indeed, this is the reason why we observed no alterations in the levels of pT180/pY182 in p38α MAPK in vivo but we did in vitro (Figs. 1 and 3). To some extent, the levels of pT180/pY182 in p38α MAPK could also be attributed to the extent of autophosphorylation by itself [64]. Therefore, we confirmed MW181-mediated p38α MAPK inhibition by assessing pATF2 both in vitro and in vivo. Optimized p38 MAPK inhibitors in this novel class of small molecules, such as MW150 [25], will also address the issue of drug candidate specificity and safety. Regardless, the results shown here added increasing support to the hypothesis that p38α MAPK is the key tau kinase under inflammatory conditions. While our previous studies suggested that MW181 may possibly target the β isoform of p38 MAPK and Nemo-like kinase (NLK) MAPK at relatively higher Ki values (322 nM and 199 nM, respectively) compared with p38α MAPK (184 nM) [26], it is the α-isoform selective inhibition of p38 MAPK that reduces tau phosphorylation and cytokine production. This conclusion is based on earlier studies, which suggested that deletion of the β isoform of p38 MAPK did not affect LPS-induced cytokine production or induce inflammation, suggesting that p38α MAPK is the primary mediator of cytokine release and inflammation [65] and that NLK is not an inducer, but a suppressor, of NF-κB activity [66], which is the primary mediator of inflammation. Taken together, these results suggest that the effects observed for MW181 in the current studies are exclusively due to targeted inhibition of the α isoform of p38 MAPK.

In the current study, MK2-deficient mice demonstrate a strong resistance to LPS-induced tau hyperphosphorylation. This suggests that suppression of IL-1β and related proinflammatory cytokine secretion is inhibited in MK2^–/–^ mice, which are otherwise induced by MK2-mediated transcription [67]. If this scenario was to be true, then one would argue that p38α MAPK should still be able to phosphorylate tau in the absence of MK2. Because IL-1β is one of the key proinflammatory cytokines important for LPS-induced p38α MAPK activation and the deficiency of MK2 leads to ablation of IL-1β production, then this suggests there is lack of a sufficient trigger for the activation of p38α MAPK following LPS injections (due to reduced expression of IL-1β and other proinflammatory cytokines). Indeed, our results show a significant reduction in the total p38 MAPK levels in MK2-deficient mice, suggesting insufficient levels of p38α MAPK in MK2^–/–^ mice. An alternative argument would be that MK2 itself might serve as a potential tau kinase. While this possibility needs further careful characterization, it is important to note that the MK2 activation via phosphorylation (at T222, S272, and T334) by p38 MAPK happens within the nucleus. Because of this, there is a very unlikely chance for the activated (and possibly p38 MAPK bound) MK2 to physically interact with tau, unless it translocates to the cytoplasm after activation. Therefore, based on current evidence, it is conceivable that reduced LPS-induced tau phosphorylation in MK2 deficient mice is due to the feed-forward abrogation of the MK2 > IL-1β > IL-1R > p38α MAPK > tau axis.

## Conclusions

The results reported in this article provide a clear validation of p38α MAPK as a target whose selective inhibition can result in improved outcomes in tauopathy models. The recent description [25] of a drug candidate that retains all of the positive attributes of MW181, but is further optimized for bioavailability and in-vivo safety, provides the means to extend the rationale presented here for tauopathies toward clinical proof of concept.

## Abbreviations

ABC: Avidin:Biotinylated enzyme Complex
AD: Alzheimer’s disease
ARG1: Arginase 1
ATF2: Activating transcription factor 2
Aβ: Amyloid beta
Cdk5: Cyclin-dependent kinase 5
CM: Conditioned media
CNS: Central nervous system
DAB: Diaminobenzidine
DIV: Days in vitro
DMSO: Dimethyl sulfoxide
ERK: Extracellular signal-related kinase
GAPDH: Glyceraldehyde 3-phosphate dehydrogenase
GPCR: G-protein-coupled receptors
GSK-3β: Glycogen synthase kinase-3β
i.p.: Intraperitoneal
ICV: Intracerebroventricular
IFNγ: Interferon-γ
IL: interleukin
IL-1R: Interleukin-1 receptor
JNK: c-Jun N-terminal kinase
LDS: Lithium dodecyl sulfate
LPS: Lipopolysaccharide
MAPT: Microtubule-associated protein tau
MARK: Microtubule affinity regulating kinase
MK2: MAPK-activated protein kinase 2
MPTP: 1-Methyl-4-phenyl-1,2,3,6-tetrahydropyridine
NFT: Neurofibrillary tangle
NF-κB: Nuclear factor kappa-light-chain-enhancer of activated B cells
NLK: Nemo-like kinase
p.o.: Per os (by mouth or via oral gavage)
p38 MAPK: p38 mitogen-activated protein kinase
PB: Phosphate buffer
PBS: Phosphate-buffered saline
PBST: PBS with 0.1% Tween
PEG: Polyethylene glycol
PFA: Paraformaldehyde
RA: Reducing agent
RGB: Red green blue
TLR4: Toll-like receptor-4
T-PER: Tissue protein extraction reagent
VEH/Veh: Vehicle
